# Honokiol‐loaded nanomicelles reprogram senescence and immune evasion in hepatocellular carcinoma via SIRT3‐mediated mitochondrial stabilization

**DOI:** 10.1002/btm2.70111

**Published:** 2026-01-18

**Authors:** Wenxing Deng, Yizhi Wu, Yisheng Yin, Jing Wang

**Affiliations:** ^1^ Department of Hepatobiliary and Pancreatic Surgery, The Central Hospital of Wuhan, Tongji Medical College Huazhong University of Science and Technology Wuhan Hubei China; ^2^ Department and Institute of Infectious Disease, Tongji Hospital, Tongji Medical College Huazhong University of Science and Technology Wuhan Hubei China; ^3^ Department of Urology, Tongji Hospital, Tongji Medical College Huazhong University of Science and Technology Wuhan Hubei China; ^4^ Institute of Organ Transplantation, Tongji Hospital, Tongji Medical College Huazhong University of Science and Technology, Key Laboratory of Organ Transplantation, Ministry of Education, NHC Key Laboratory of Organ Transplantation, Key Laboratory of Organ Transplantation, Chinese Academy of Medical Sciences, Organ Transplantation Clinical Medical Research Center of Hubei Province Wuhan Hubei China

**Keywords:** cellular senescence, hepatocellular carcinoma, nanomicelles, tumor immune microenvironment

## Abstract

Hepatocellular carcinoma (HCC) is a major cause of cancer‐related deaths. Advanced‐stage patients face poor prognosis due to chemotherapy resistance and an immunosuppressive tumor microenvironment (TME). Cellular senescence, marked by the senescence‐associated secretory phenotype, promotes tumor progression and immune evasion. Although honokiol (HKL) shows strong antitumor activity, its clinical use is limited by poor solubility, rapid clearance, and low bioavailability. Here, we report HKL‐loaded poly(ethylene glycol)‐poly(ε‐caprolactone)‐poly(ethylene glycol) triblock copolymer nanomicelles (HKL‐nm) as a multifunctional nanotherapeutic platform. HKL‐nm exhibited a uniform spherical shape with a diameter of 60.93 ± 5.7 nm, near‐neutral charge (−0.28 ± 0.1 mV), and high encapsulation efficiency (85.9 ± 4.9%). It enabled sustained drug release (70.04 ± 6.2% over 200 h) and significantly improved oral pharmacokinetics (area under the curve increased 6.26‐fold and *C*_max_ increased 4.06‐fold). Specifically, the HKL drug concentration at the tumor site was enhanced by 3.52‐fold. Mechanistically, HKL‐nm suppressed senescence markers (p53, p16, and p21) and senescence‐associated β‐galactosidase positivity via a Sirtuin 3‐dependent pathway, inhibiting cytoplasmic mitochondrial DNA leakage and cGAS‐STING signaling. In Hepa1‐6 cells xenografts, combination therapy with HKL‐nm and the senolytic cocktail dasatinib + quercetin achieved tumor volume reduction, with transcriptomic analysis validating enrichment of immune activation pathways. This was accompanied by enhanced infiltration of CD8^+^ cytotoxic T cells and mature dendritic cells, coupled with profound suppression of myeloid‐derived suppressor cells. By integrating nanodelivery, senescence modulation, and immuno‐oncology, HKL‐nm represents a promising strategy to overcome therapeutic resistance in HCC, providing a preclinical basis for translation to solid tumors.

## Graphical abstract

(a) Schematic diagram illustrating the preparation process of honokiol (HKL)‐loaded poly(ethylene glycol)‐poly(ε‐caprolactone)‐poly(ethylene glycol) nanomicelles. (b) Anti‐tumor mechanisms: HKL‐nm induces apoptosis, suppresses senescence‐associated secretory phenotype, and stabilizes mitochondria to prevent mitochondrial DNA release. This blocks cGAS‐STING activation, thereby reprogramming the tumor microenvironment.

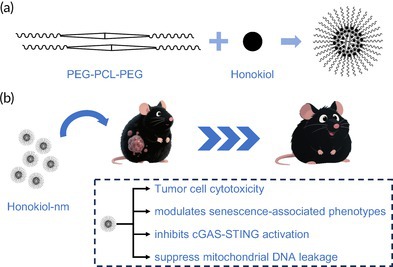

**Abbreviations**
BCA: bicinchoninic acid
cGAS‐STING: cyclic guanosine monophosphate‐adenosine monophosphate synthas‐stimulator of interferon genes
DMSO: dimethylsulfoxide
EDTA: ethylene diamine tetraacetic acid
ELISA: enzyme‐linked immunosorbent assay
FDR: false discovery rate
H&E: hematoxylin‐eosin staining
HPLC: high performance liquid chromatography
HRP: horseradish peroxidase
IFN‐γ: interferon γ
IL‐1β: interleukin‐1β
IL‐6: interleukin‐6
IL‐16: interleukin‐16
KEGG: kyoto encyclopedia of genes and genomes
MWCO: molecular weight cut off
NF‐κB: nuclear factor kappa B
Nrf2/HO‐1: nuclear factor erythroid 2‐related factor 2‐heme oxygenase 1
PBST: phosphate buffered saline‐tween
PDI: polymer dispersity index
PMSF: phenylmethanesulfonyl fluoride
qRT‐PCR: real‐time quantitative reverse transcription polymerase chain reaction
Ras‐RAF: rat sarcoma‐Raf protein kinase
SDS‐PAGE: sodium dodecyl sulfate polyacrylamide gel electrophoresis
TFAM: transcription factor A
TMB: 3,3',5,5'‐tetramethylbenzidine
TNF‐α: tumor necrosis factor α
TUNEL: terminal‐deoxynucleoitidyl transferase mediated nick end labeling
USP: United States Pharmacopoeia
WB: Western Blot

**Translational Impact Statements**

This study delivers a multifunctional nanotherapeutic platform that overcomes the critical bioavailability limitations of honokiol (HKL) for treating hepatocellular carcinoma (HCC). By encapsulating HKL in poly(ethylene glycol)‐poly(ε‐caprolactone)‐poly(ethylene glycol) nanomicelles, HKL‐nm achieves sustained release, significantly enhances oral pharmacokinetics, and boosts drug delivery to the tumor site. Crucially, HKL‐nm mechanistically targets the immunosuppressive and pro‐tumorigenic senescence‐associated secretory phenotype within the tumor microenvironment via a Sirtuin 3‐dependent pathway, inhibiting cytoplasmic mitochondrial DNA leakage and blocking cGAS‐STING signaling. HKL‐nm enables potent combination therapy with senolytics (dasatinib + quercetin), achieving significant tumor regression in preclinical models by synergistically eliminating senescent cells, reversing immunosuppression, enhancing infiltration of cytotoxic CD8^+^ T cells and mature dendritic cells, and suppressing myeloid‐derived suppressor cells. Collectively, HKL‐nm provides a promising preclinical strategy to overcome therapeutic resistance in advanced HCC and establishes a translatable platform adaptable for treating other solid tumors characterized by senescence and immunosuppression.

## 1 INTRODUCTION

Hepatocellular carcinoma (HCC) remains a formidable global health challenge, ranking as the fourth leading cause of cancer‐related mortality worldwide.^1^ Despite advances in surgical resection and ablation, the prognosis for advanced patients is often compromised by high recurrence rates and resistance to conventional chemotherapy or immunotherapy.^2^ A critical barrier to effective treatment is the highly immunosuppressive tumor microenvironment (TME), which protects residual tumor cells from immune surveillance.^3^

Cellular senescence, a state of stable cell cycle arrest, has emerged as a pivotal regulator of the TME.^4^ The accumulation of senescent cells gives rise to a pro‐tumorigenic and immunosuppressive environment via the senescence‐associated secretory phenotype (SASP).^5^, ^6^ Recent mechanistic studies have identified the cGAS‐STING signaling pathway as a central driver of SASP. Specifically, mitochondrial dysfunction in senescent cells leads to the leakage of mitochondrial DNA (mtDNA) into the cytoplasm, which triggers aberrant cGAS‐STING activation and sustains the chronic inflammatory SASP.^7^ Consequently, targeting the mitochondrial‐cGAS‐STING axis to inhibit SASP represents a promising avenue to overcome immune evasion.^8^

Honokiol (HKL), a natural biphenolic compound derived from *Magnolia officinalis*, has been extensively studied for its role as a natural agonist of Sirtuin 3 (SIRT3).^9^ Several studies demonstrate its ability to induce cell cycle arrest and apoptosis, and to inhibit angiogenesis by modulating NF‐κB, Ras–RAF, and Nrf2/HO‐1 signaling pathways.^10^ Notably, HKL has shown efficacy in suppressing cancer cell migration and invasion and in sensitizing tumors to immune checkpoint inhibitors.^11^ However, its clinical translation is hindered by its poor aqueous solubility, rapid metabolism, and low oral bioavailability.

Nanotechnology‐based drug delivery systems (DDS) offer a promising solution.^12^ Amphiphilic copolymers, such as poly(ethylene glycol)‐poly(ε‐caprolactone)‐poly(ethylene glycol) (PEG‐PCL‐PEG) triblock copolymer nanomicelles, present a biodegradable and versatile platform. The hydrophobic PCL core efficiently encapsulates HKL, while the hydrophilic PEG shell enhances solubility and minimizes non‐specific interactions, thereby forming a biocompatible platform with an adjustable drug‐loading capacity and extended circulation time.^13^ Such nanosystems have been shown to improve drug retention in tumors by three‐ to five‐fold compared to free drugs.^14^, ^15^

Building on these advancements, in this study, we developed a HKL‐loaded PEG‐PCL‐PEG nanomicellar system (HKL‐nm) to overcome the pharmacokinetic barriers of free HKL and amplify its antitumor efficacy. We hypothesized that HKL‐nm could disrupt tumor cell senescence by targeting the SASP and mtDNA‐driven inflammatory pathways. We further explored the synergistic potential of combining HKL‐nm with a senolytic regimen (dasatinib + quercetin, DQ) to reprogram the immunosuppressive TME and enhance cytotoxic immunity.

Our work introduces several key innovations. HKL‐nm enables sustained drug release and targets cellular senescence through SIRT3‐dependent mitochondrial stabilization. Mechanistically, HKL‐nm treatment suppressed cGAS‐STING pathway activation and SASP by preventing mtDNA leakage into the cytoplasm. The combination of HKL‐nm and DQ not only alleviates the burden of senescent cells but also enhances CD8^+^ T cell infiltration while suppressing myeloid‐derived suppressor cells (MDSCs), thereby remodeling the TME into an immunogenic state. By bridging nanotechnology, senescence biology, and immuno‐oncology, this study provides a transformative paradigm for HCC treatment, offering a roadmap for the rational design of natural product‐based nanotherapeutics in solid tumors.

## 2 RESULTS

### 2.1 Structural characterization and drug delivery performance of HKL‐nm

**FIGURE 1:**
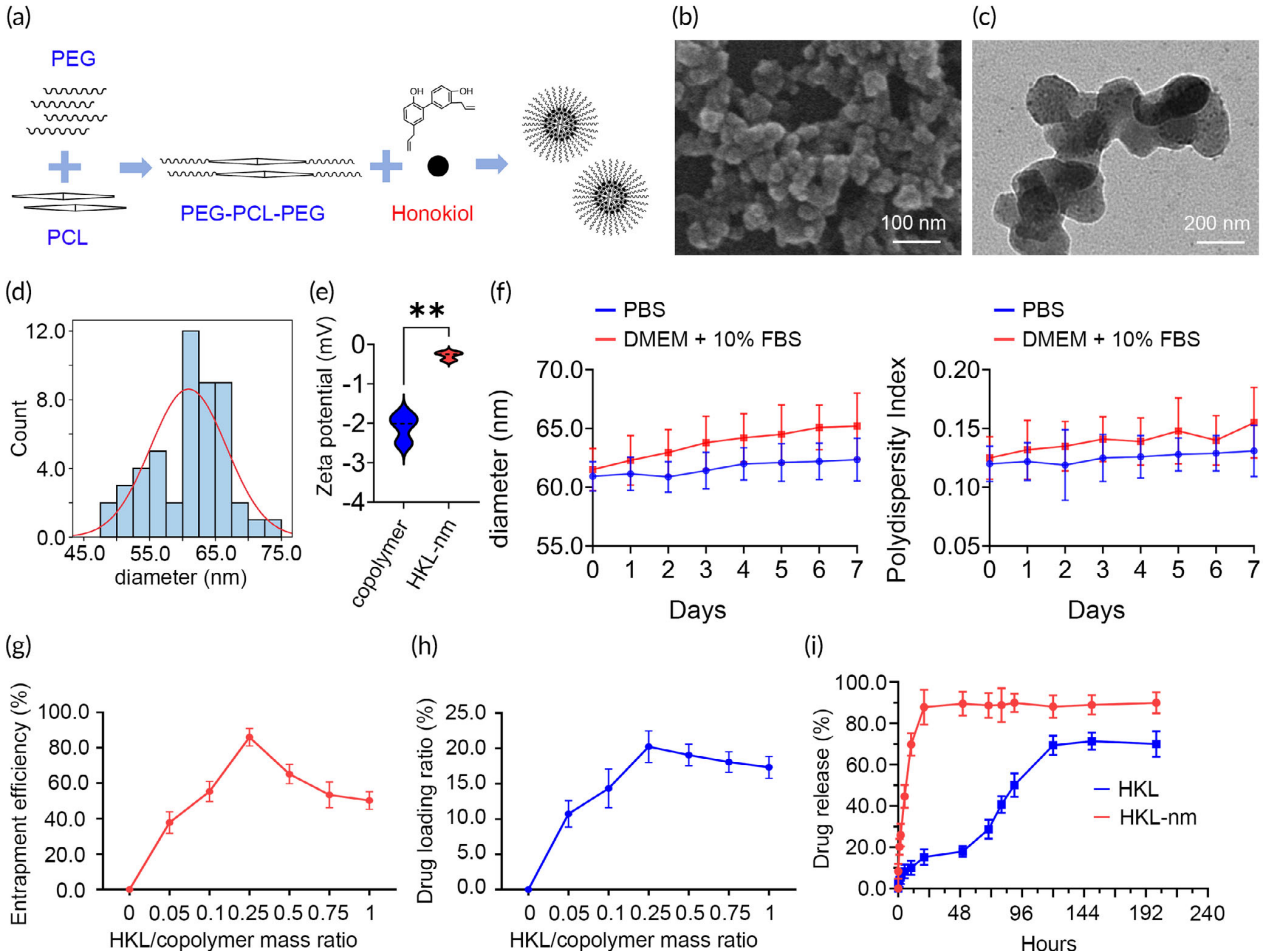
Preparation and characterization of honokiol‐loaded poly(ethylene glycol)‐poly(ε‐caprolactone)‐poly(ethylene glycol) triblock copolymer nanomicelles (HKL‐nm). (a) Schematic illustration of HKL‐nm preparation via oil‐in‐water self‐assembly; (b) scanning electron microscopy image of HKL‐nm, scale bar: 100 nm; (c) transmission electron microscopy image of HKL‐nm, scale bar = 200 nm; (d) Size distribution histogram analyzed by dynamic light scattering; (e) zeta potential of blank micelles and HKL‐nm; (f) the changes in diameter and polydispersity index of HKL‐nm were monitored during incubation in phosphate‐buffered saline (PBS) (pH 7.4) or Dulbecco's Modified Eagle Medium (DMEM) containing 10% fetal bovine serum (FBS) at 37°C for 7 days. (g) Effect of drug/carrier mass ratios on encapsulation efficiency; (h) effect of drug/carrier mass ratios on drug‐loading capacity; (i) in vitro sustained‐release profile of HKL‐nm. Data are presented as mean ± standard deviation (*n* = 3). **p < 0.01.

The preparation process and structure of HKL‐nm were illustrated in Figure 1a. The water‐in‐oil self‐assembly technique effectively produced spherical nanoparticles exhibiting consistent morphology. In an aqueous solution, the amphiphilic PEG‐PCL‐PEG triblock copolymer forms micelles with a core–shell architecture. Within this arrangement, the core is formed by hydrophobic PCL chains, whereas the shell is made up of hydrophilic PEG chains. Leveraging the hydrophobic interactions of PCL, combined with the interaction between water and the hydrophilic PEG layer, it is possible to efficiently encapsulate hydrophobic HKL inside the core region. Scanning electron microscopy (SEM) imaging (Figure 1b) revealed HKL‐nm with smooth surfaces, while transmission electron microscopy (TEM) analysis (Figure 1c) confirmed their core–shell architecture. Dynamic light scattering (DLS) measurements (Figure 1d) demonstrated a narrow size distribution of 60.93 ± 5.7 nm (PDI: 0.12 ± 0.03). HKL‐nm exhibited a substantially reduced negative zeta potential (−0.28 ± 0.1 mV) compared to blank carriers (−2.1 ± 0.36 mV, *p* < 0.01. Figure 1e). This suggests that the surface charge of the HKL‐nm can be considered nearly neutral. To evaluate the potential for in vivo application, the stability of HKL‐nm was also assessed in physiological conditions (phosphate‐buffered saline [PBS], pH 7.4) and serum‐containing simulants (10% fetal bovine serum [FBS]) at 37°C. As shown in Figure 1f, the HKL‐nm displayed excellent stability profiles in both media. Over a period of 7 days, the mean hydrodynamic diameter exhibited minimal variation, maintaining a size range of 60–65 nm. Crucially, the PDI values remained consistently low (<0.2), indicating a uniform size distribution and the absence of particle aggregation or disassembly. This robust stability in serum‐containing environments suggests that the dense PEG corona effectively provides steric hindrance, shielding the nanoparticles from non‐specific protein adsorption and destabilization. Systematic optimization of drug/carrier mass ratios revealed a positive correlation between polymer content and encapsulation efficiency (Figure 1g). The maximum encapsulation efficiency reached 85.9 ± 4.9% at a 1:4 ratio, whereas maximum drug‐loading capacity was 20.2 ± 2.2% under the same conditions (Figure 1h). In vitro release experiments (Figure 1i) revealed that free HKL was released quickly, achieving 87.9 ± 8.4% of the total quantity within the initial 12 h. Conversely, HKL released from the micellar system displayed prolonged release behavior, with an initial release of 15.2 ± 3.7% during the first 12 h, followed by a slower release phase, resulting in a total cumulative release of 70.04 ± 6.2% over 200 h. This extended release profile of HKL is primarily due to its encapsulation within the micelle core. As a result, the designed copolymeric micelles provide a highly promising platform for time‐controlled release of hydrophobic drugs, contributing to the achievement of diverse therapeutic goals.

### 2.2 Enhanced biocompatibility and pharmacokinetic optimization of HKL‐nm

**FIGURE 2:**
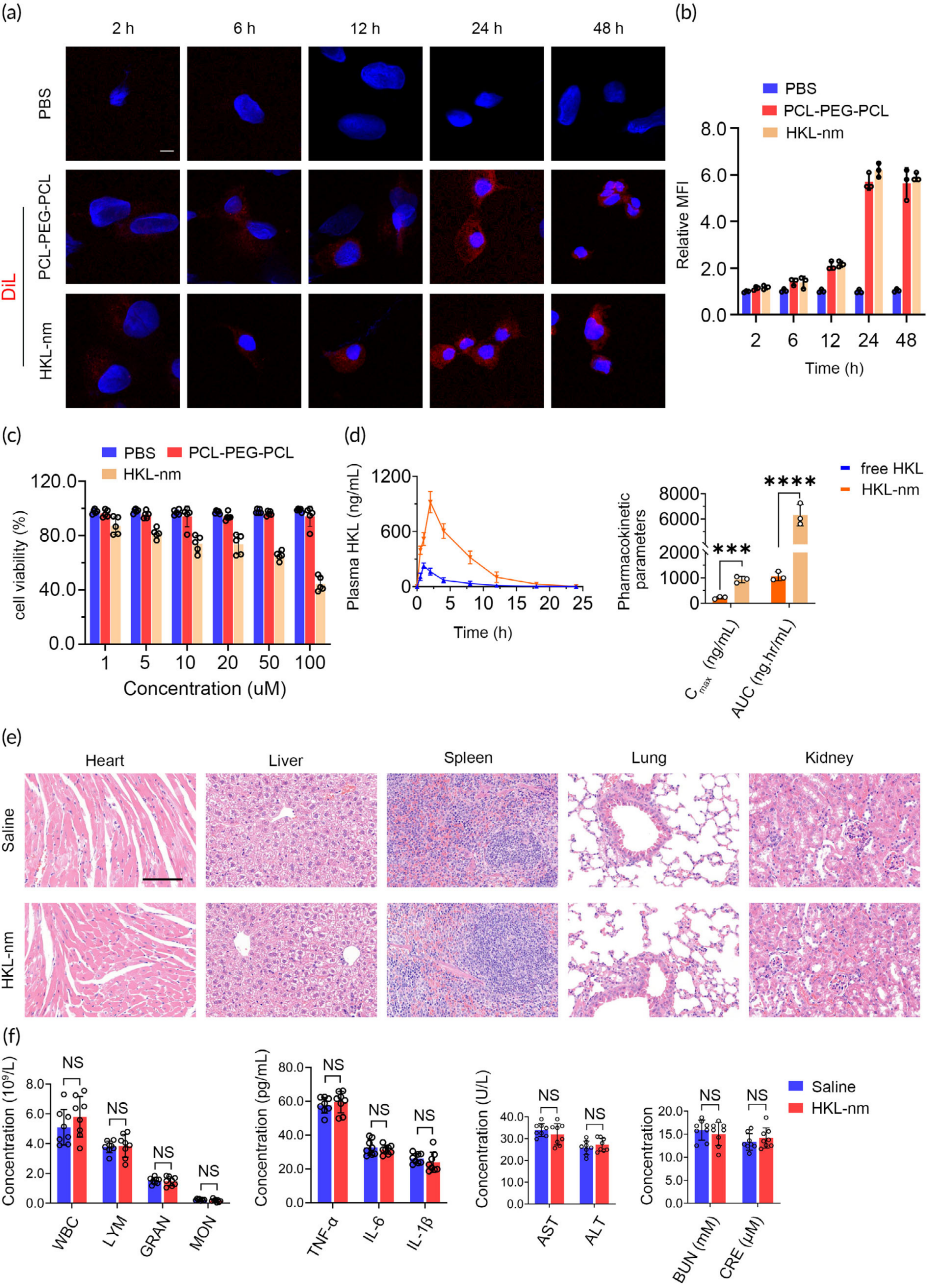
Biocompatibility and pharmacodynamic evaluation of honokiol‐loaded poly(ethylene glycol)‐poly(ε‐caprolactone)‐poly(ethylene glycol) (PEG‐PCL‐PEG) triblock copolymer nanomicelles (HKL‐nm). (a) Time‐dependent cellular uptake: Fluorescence microscopy images and (b) quantitative analysis of fluorescence intensity (right) at 2, 6, 12, 24, and 48 h post‐incubation (culture conditions: Dulbecco's Modified Eagle Medium [DMEM] + 10% fetal bovine serum, HKL equivalent dose: 50 μM; scale bar = 20 μm). (c) Cytotoxicity assessment via CCK‐8 assay under increasing HKL concentrations (1–100 μg/mL, 24 h incubation). (d) Pharmacokinetic profiles: Plasma HKL concentration‐time curves after oral administration of free HKL and HKL‐nm (100 mg/kg, blood sampling intervals: 0–24 h; detection limit: 0.1 ng/mL). Pharmacokinetic parameters: Comparative *C*_max_ and AUC_0–*t*_ values; (e) Representative H&E stained images of the major organs from mice following long‐term administration of HKL‐nm or saline (40 mg/kg, once weekly for 1 month, intravenous injection). (f) Hematological parameters, inflammatory cytokine levels, liver function indicators, and kidney function indicators of the long‐term treated mice in different treatment groups. Data are presented as mean ± SD. *n* = 8. ***p < 0.001,****p < 0.0001, ALT, alanine aminotransferase; AST, aspartate aminotransferase; AUC, area under the curve; BUN, blood urea nitrogen; CRE, serum creatinine; GRAN, neutrophil count; LYM, lymphocyte count; MON, monocyte count; ns, no significance; PBS, phosphate‐buffered saline; WBC, white blood cell count.

The HKL‐nm exhibited improved cellular uptake and better biocompatibility. As depicted in Figure 2a, our results demonstrated that HepG2 cells were capable of efficiently internalizing HKL‐nm and revealing that uptake reached equilibrium at 24 h (Figure 2b). The PEG‐PCL‐PEG copolymer exhibited negligible cytotoxic effects after being successfully internalized by tumor cells. In contrast, treatment with HKL‐nm resulted in a dose‐dependent decrease in cell viability. Notably, HKL‐nm markedly reduced cell viability at a concentration of 100 μg/mL (Figure 2c). The in vivo safety of HKL‐nm was evaluated following short‐term (24 h) and long‐term (28 days) administration. Mice received either a single dose tail vein injection (100 mg/kg) or repeated therapeutic doses (40 mg/kg, once weekly for 1 month), followed by blood collection and histological examination. The results show no significant pathological abnormalities in the treatment groups compared to the PBS control (Figures 2e and S1A). No significant differences in hematological parameters, inflammatory cytokine levels, liver function indicators, and kidney function indicator were observed between the saline and HKL‐nm groups (Figures 2f and S1B). Overall, HKL‐nm did not induce significant systemic toxicity.

HKL‐nm showed significantly improved oral pharmacokinetics compared to the free HKL. The *C*_max_ and area under the curve (AUC) values were markedly higher in the HKL‐nm groups. Specifically, at a dose of 100 mg/kg, the *C*_max_ was increased by 4.3‐fold (217.3 ± 51.92 vs. 935.3 ± 124.3 ng/mL, *p* < 0.001) and the AUC was elevated by 6.05‐fold (1062 ± 175.2 vs. 6305 ± 857.2 ng h/mL, *p* < 0.0001) compared to the free drug (Figure 2d). Thus, nanomicellar formulations greatly enhance HKL's oral bioavailability.

### 2.3 Senescence suppression by HKL‐nm in hepatocellular carcinoma cells

**FIGURE 3:**
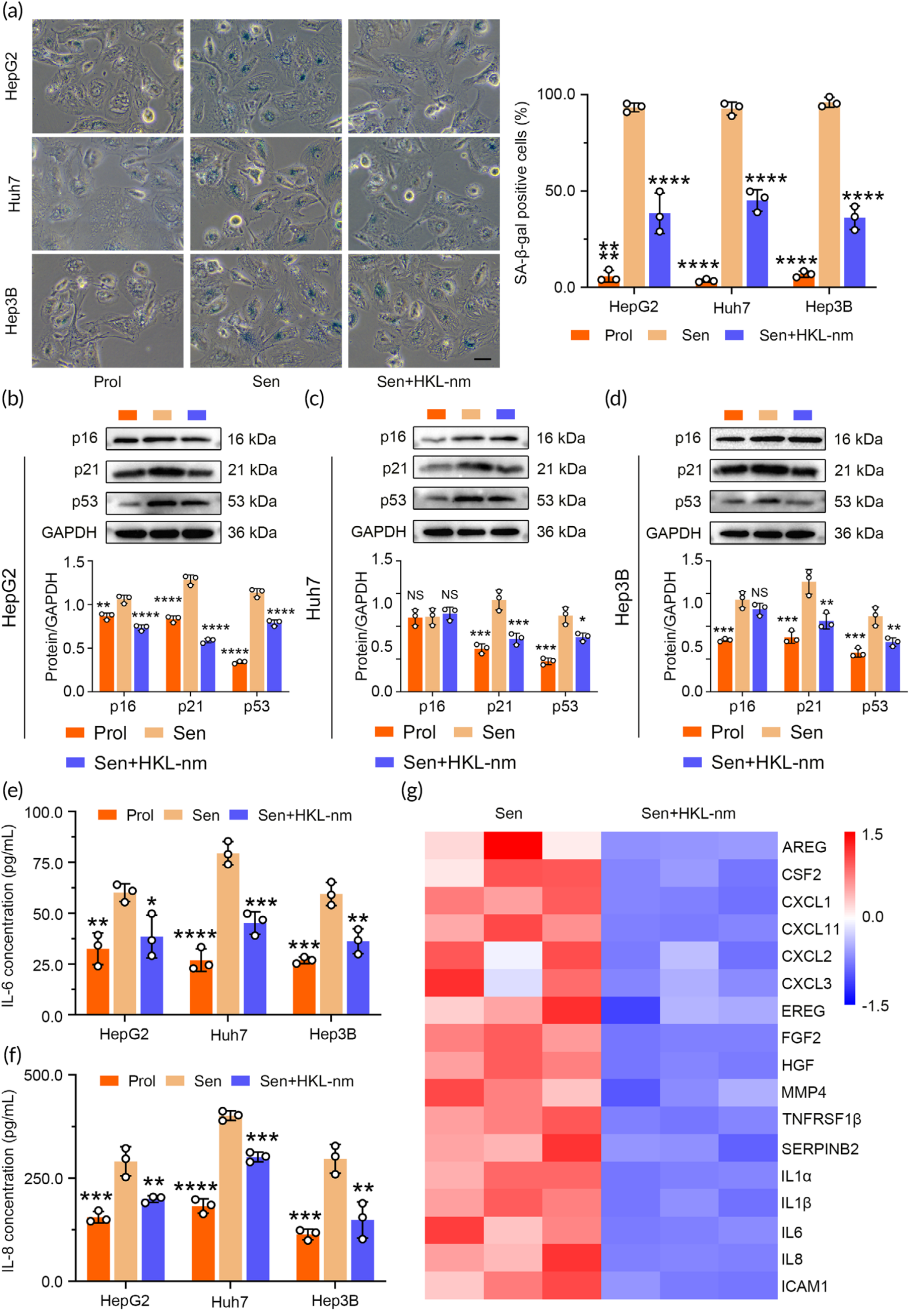
Honokiol‐loaded poly(ethylene glycol)‐poly(ε‐caprolactone)‐poly(ethylene glycol) triblock copolymer nanomicelles (HKL‐nm) modulates senescence‐associated phenotypes across hepatocellular carcinoma cell models. (a) Senescence‐associated β‐galactosidase (SA‐β‐gal) staining in three HCC cell lines (Hep3B, Huh7, HepG2) under proliferating (Prol), senescent (Sen), and Sen + HKL‐nm treated conditions. Left: Representative bright‐field images (scale bar = 50 μm). Right: Quantification of SA‐β‐gal‐positive cells (vs. Sen group). (b–d) Western blot analysis of senescence markers (p16, p21, and p53) with GAPDH normalization in (b) HepG2, (c) Huh7, and (d) Hep3B cells. Top: Immunoblot bands (antibody dilutions: 1:1000 for p16/p21/p53, 1:5000 for GAPDH). Bottom: Densitometric quantification (vs. Sen group). (e, f) ELISA quantification of (e) IL‐6 and (f) IL‐8 in conditioned media from three hepatocellular carcinoma lines (vs. Sen group). (g) RNA sequencing heatmap of senescence‐associated secretory phenotype (SASP)‐related genes in Sen‐HepG2 cells with/without HKL‐nm treatment (|log_2_FC| >1, FDR < 0.05). *p < 0.05, **p < 0.01, ***p < 0.001, ****p < 0.0001, ns, no significance.

HKL‐nm treatment markedly inhibited senescence‐associated characteristics in HCC cells. Senescence‐associated β‐galactosidase (SA‐β‐gal) staining (Figure 3a) indicated that HKL‐nm decreased the proportion of senescent cells in all three HCC lines. Specifically, in HepG2 cells, the senescent population was reduced from 38.49 ± 10.52% to 3.41 ± 1.26% (*p* < 0.0001); in Huh7 cells, it decreased from 92.75 ± 3.38% to 45.15 ± 5.55% (*p* < 0.0001); and in Hep3B cells, it dropped from 96.08 ± 2.56% to 36.15 ± 6.11% (*p* < 0.0001). WB analysis (Figure 3b–d) demonstrated a consistent downregulation of senescence markers. In HepG2 cells, HKL‐nm treatment led to a significant decrease in p21 protein expression (*p* < 0.0001), along with reductions in p16 (*p* < 0.0001) and p53 (*p* < 0.0001). Comparable patterns of suppression were observed in both Huh7 and Hep3B models. The anti‐senescence effects also extended to secretory phenotypes. ELISA measurements (Figure 3e,f) revealed that HKL‐nm treatment reduced IL‐6 and IL‐8 secretion compared to untreated senescent cells. For instance, in HepG2 cells, IL‐6 levels decreased from 60.08 ± 4.37 pg/mL to 38.49 ± 10.52 pg/mL (*p* < 0.05), and IL‐8 levels dropped from 290.5 ± 35.23 pg/mL to 199.1 ± 8.06 pg/mL (*p* < 0.05). RNA sequencing (RNA‐seq) analysis (Figure 3g) further identified HKL‐nm‐mediated suppression of SASP‐related genes in Sen‐HepG2 cells (|log2FC| >1), including proinflammatory factors such as IL1α, IL1β, IL6, IL8, CXCL1, CXCL11, CXCL2, CXCL3, and matrix metalloproteinases like MMP4.

### 2.4 HKL‐nm treatment suppressed cGAS‐STING pathway activation through SIRT3‐dependent mitochondrial genome stabilization

**FIGURE 4:**
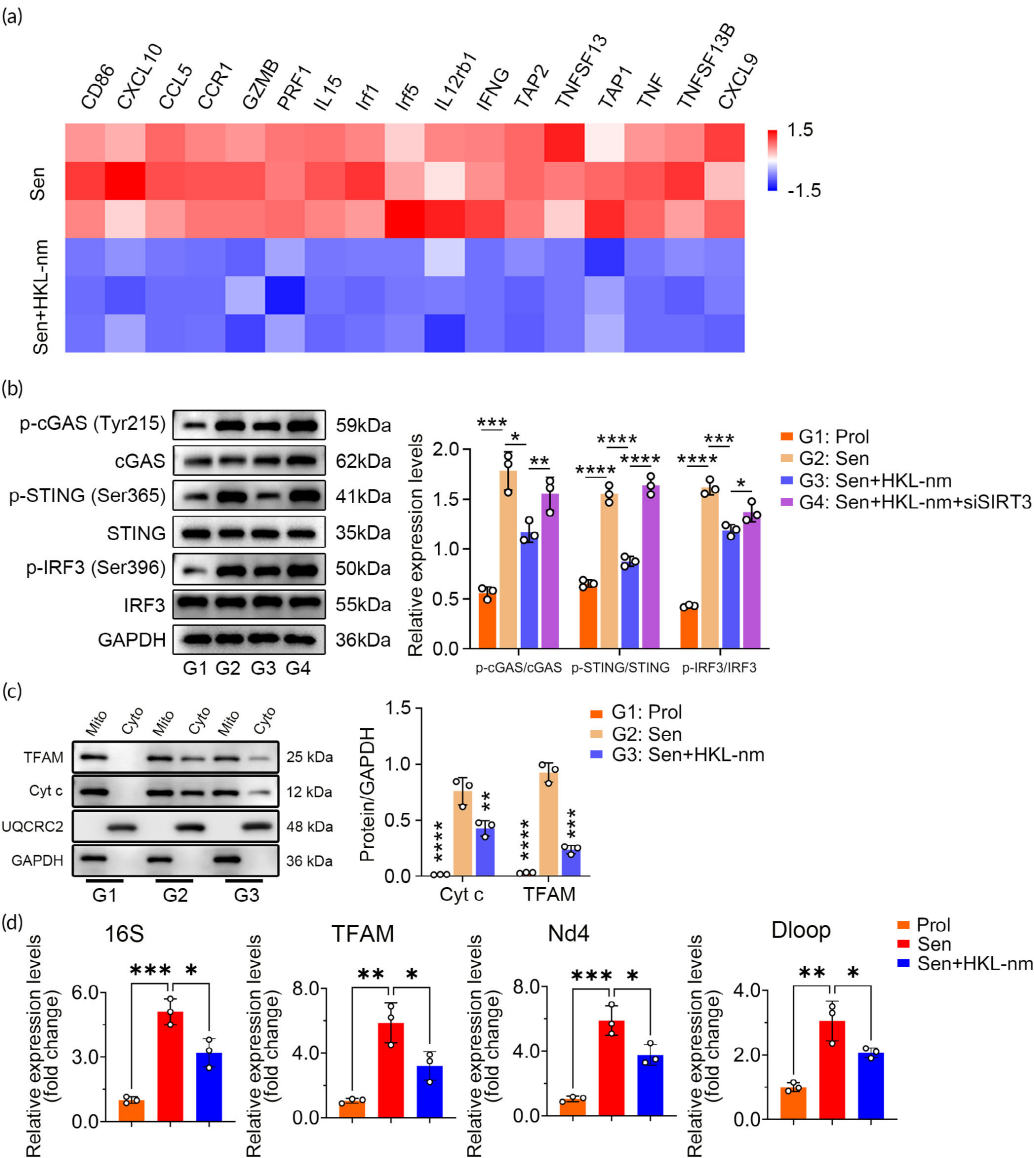
Honokiol‐loaded poly(ethylene glycol)‐poly(ε‐caprolactone)‐poly(ethylene glycol) triblock copolymer nanomicelles (HKL‐nm) inhibits cGAS‐STING activation via mitochondrial DNA leakage suppression. (a) RNA sequencing (RNA‐seq) heatmap of cGAS‐STING signaling‐related genes. (b) Western blot analysis of cGAS‐STING pathway activation: Phosphorylation levels of cGAS (Tyr215), STING, and IRF3 (Ser396) under proliferating (Prol), senescent (Sen), Sen + HKL‐nm, and Sen + HKL‐nm + siSIRT3 conditions (1:1000 for phospho‐proteins, 1:5000 for GAPDH). (c) Cytosolic mitochondrial DNA leakage assessed by cytoplasmic TFAM and cytochrome c (Cyt c) levels via subcellular fractionation (mitochondrial marker: UQCRC2; cytosolic marker: GAPDH). (d) qRT‐PCR quantification of cytoplasmic mitochondrial DNA fragments (16S rRNA, TFAM, Nd4, D‐loop) normalized to 18S rRNA. *p < 0.05, **p < 0.01, ***p < 0.001, ****p < 0.0001, ns, no significance.

The cGAS‐STING pathway plays a key role in activating an immune response to cytoplasmic chromatin fragments, driving inflammation. This pathway promotes tumorigenesis in various contexts, making it a potential cancer therapy target. HKL, a natural compound, modulates the cGAS‐STING pathway. We investigated whether HKL‐nm regulates this pathway to suppress SASP. RNA‐seq profiling (Figure 4a) revealed significant downregulation of cGAS‐STING signaling components and downstream inflammatory genes in HKL‐nm‐treated senescent HepG2 cells (log2FC <−1.5, FDR <0.05). At the molecular level, we examined the expression of key signaling molecules involved in cGAS‐STING activation. WB analysis (Figure 4b) demonstrated that HKL‐nm reduced phosphorylation of cGAS (Tyr215), STING, and IRF3 (Ser396) compared to untreated senescent cells (*p* < 0.01). Critically, SIRT3 knockdown (siSIRT3) abolished these effects, upregulated phosphoprotein levels to senescence baseline, confirming SIRT3 as the mechanistic target. Mechanistically, HKL‐nm prevented mtDNA leakage into the cytoplasm. Subcellular fractionation assays (Figure 4c) showed HKL‐nm reduced cytosolic TFAM (*p* < 0.01) and cytochrome c (*p* < 0.001), indicating restored mitochondrial membrane integrity. qRT‐PCR quantification (Figure 4d) further confirmed decreases in cytoplasmic mtDNA fragments (16S rRNA, D‐loop, Nd4) after HKL‐nm treatment, suggesting attenuated mtDNA‐driven cGAS activation.

### 2.5 HKL‐nm treatment exerted strong antitumor effects in hepatocellular carcinoma xenograft models, with improved outcomes when combined with the DQ senolytic regimen

**FIGURE 5:**
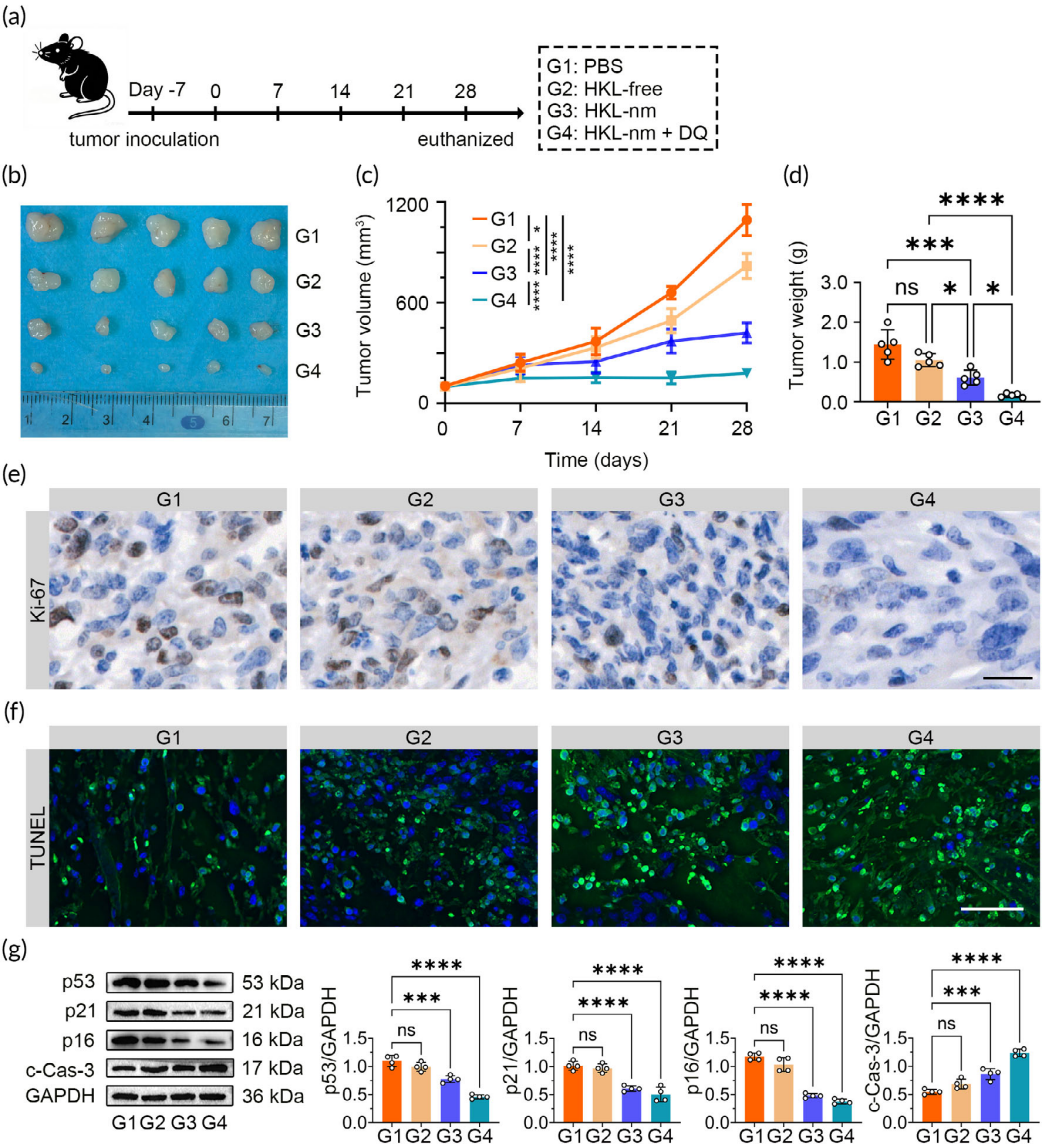
Honokiol‐loaded poly(ethylene glycol)‐poly(ε‐caprolactone)‐poly(ethylene glycol) triblock copolymer nanomicelles (HKL‐nm) suppresses tumor growth and modulates senescence‐apoptosis axis in hepatocellular carcinoma xenografts. (a) Schematic of subcutaneous xenograft experiment (Hepa1‐6 cells in C57BL/6J mice. (b) Macroscopic view of excised tumors. (c) Tumor growth curves. (d) Terminal tumor weight comparison. (e) Ki‐67 immunohistochemical staining. scale bar = 20 μm. (f) TUNEL staining scale bar = 50 μm. (g) Western blot analysis of senescence markers (p16/p21/p53) and apoptosis marker cleaved caspase‐3 (antibody dilutions: 1:1000 target proteins, 1:5000 GAPDH). *p < 0.05, ***p < 0.001, ****p < 0.0001, ns, no significance; PBS, phosphate‐buffered saline.

Schematic of subcutaneous xenograft experiment was shown in Figure 5a. HKL‐nm monotherapy significantly suppressed tumor growth, reducing final tumor volume by 67% compared to the control group (326.15 ± 38 mm^3^ vs. 989.52 ± 45 mm^3^, *p* < 0.001), whereas free HKL achieved only 28% inhibition (712.45 ± 92 mm^3^, *p* < 0.05, compared to the PBS group). The combination therapy exhibited synergistic effects, further decreasing tumor volume to 218 ± 25 mm^3^ (*p* < 0.01 vs. HKL‐nm monotherapy) (Figure 5b,c). Terminal tumor weight analysis (Figure 5d) corroborated these findings: HKL‐nm reduced tumor mass by 64% (0.82 ± 0.11 g vs. control 2.28 ± 0.23 g, *p* < 0.0001), whereas the combination group showed an 81% reduction (0.43 ± 0.07 g, *p* < 0.01 vs. HKL‐nm). Immunohistochemistry revealed that HKL‐nm + DQ suppressed proliferative activity most effectively, lowering Ki‐67 positivity to 12.6 ± 1.8% (vs. 48.3 ± 4.2% in control, *p* < 0.0001; Figure 5e). Mechanistically, HKL‐nm treatment induced apoptosis while simultaneously suppressing senescence in tumor tissues. Western blot analysis (Figure 5g) revealed a significant increase in cleaved caspase‐3, an established marker of apoptosis, in the HKL‐nm group compared to the control group (*p* < 0.001). These findings were corroborated by TUNEL staining results (Figure 5f), which demonstrated consistent patterns of apoptosis induction. The drug concentration at the tumor site was also enhanced by 3.52‐fold (64.63 ± 22.52 vs. 227.5 ± 79.29 ng/mL, *p* < 0.05) (Figure S2). Additionally, there were significant reductions in senescence‐associated markers, including p53 (*p* < 0.001), p21 (*p* < 0.0001), and p16 (*p* < 0.0001). The combination therapy amplified these effects, achieving more dramatic p53/p16/p21 suppression (Figure 5g).

### 2.6 Immune microenvironment reprogramming and enhanced cytotoxic immunity via HKL‐nm combined with the DQ senolytic regimen

**FIGURE 6:**
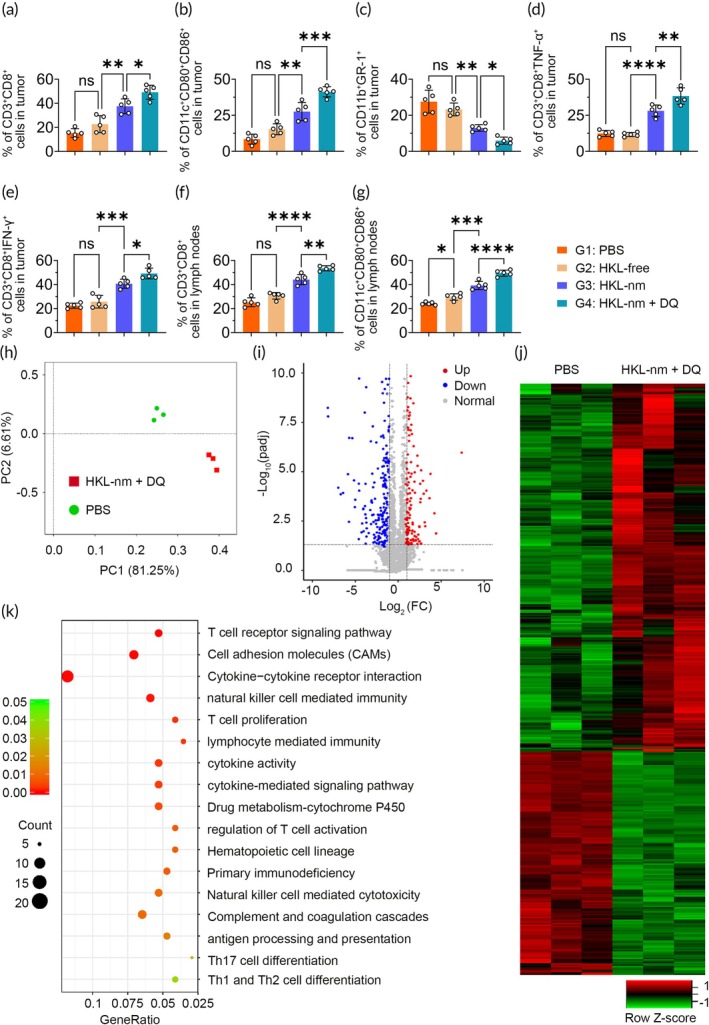
Honokiol‐nanomicelles (HKL‐nm) reprograms the tumor immune microenvironment and activates systemic antitumor immunity. (a) Tumor‐infiltrating CD8^+^ T cell percentages analyzed by flow cytometry. (b) Mature dendritic cells (DCs: CD80^+^CD86^+^) in tumor tissues. (c) Myeloid‐derived suppressor cells (CD11b^+^Gr‐1^+^) infiltration. (d, e) Functional CD8^+^ T cells producing (d) TNF‐α and (e) IFN‐γ. (f, g) Lymph node immune profiling: (f) CD8^+^ T cells and (g) mature DCs. (h–j) RNA‐seq analysis of HKL‐nm + dasatinib + quercetin (DQ)‐treated tumors: (h) PCA plot, (i) volcano plot (threshold: |log2FC| >1, *p* < 0.05), (j) Differentially expressed gene (DEG) clustering heatmap. (k) KEGG enrichment of DEGs. *p < 0.05, **p < 0.01, ***p < 0.001, ****p < 0.0001, ns, no significance; PBS, phosphate‐buffered saline.

HKL‐nm alone or combined with the DQ regimen profoundly remodeled the tumor immune microenvironment, potentiating antitumor immunity. Flow cytometry analysis revealed that HKL‐nm monotherapy increased tumor‐infiltrating CD8^+^ T cells compared to the free HKL (37.5 ± 6.14% vs. 22.64 ± 7.05%, *p* < 0.01), while the combination therapy further elevated this to 49.14 ± 5.8% (*p* < 0.05) (Figure 6a). Concurrently, mature DCs surged to 41.32 ± 3.5% in the combination group (vs. 27.56 ± 6.35% in HKL‐nm, *p* < 0.001; 27.56 ± 6.35% in HKL‐nm vs. 15.32 ± 3.78% in free HKL, *p* < 0.001), indicating enhanced antigen presentation (Figure 6b). The therapies also suppressed immunosuppressive elements: MDSCs decreased in the HKL‐nm + DQ group (5.9 ± 1.9% in vs. 12.98 ± 1.84% in HKL‐nm, *p* < 0.05; 12.98 ± 1.84% in HKL‐nm vs. 23.24 ± 3.5% in free HKL, *p* < 0.001. Figure 6c). Functional assays demonstrated that HKL‐nm + DQ synergistically boosted TNF‐α^+^CD8^+^ T cells (38.38 ± 5.6% vs. 28.08 ± 4.03% in HKL‐nm, *p* < 0.01; 28.08 ± 4.03% in HKL‐nm vs. 11.68 ± 1.44% in free HKL, *p* < 0.001; Figure 6d) and IFN‐γ^+^CD8^+^ T cells (49.14 ± 4.47% vs. 40.64 ± 3.74% in HKL‐nm, *p* < 0.05; 40.64 ± 3.74% in HKL‐nm vs. 25.68 ± 5.2% in free HKL, *p* < 0.001; Figure 6e), confirming effector T cell activation. Systemic immune activation was also evident in tumor‐draining lymph nodes, where CD8^+^ T cells increased to 25.3 ± 1.6% in HKL‐nm + DQ (53.46 ± 2.1% vs. 44.08 ± 4.2% in HKL‐nm, *p* < 0.01; 44.08 ± 4.2% in HKL‐nm vs. 30.62 ± 2.7% in free HKL, *p* < 0.0001; Figure 6f) and mature DCs reached 49.46 ± 2.5% in the combination group (vs. 39.16 ± 3.4% in HKL‐nm, *p* < 0.001; 39.16 ± 3.4% in HKL‐nm vs. 29.58 ± 2.7% in free HKL, *p* < 0.0001 Figure 6g). RNA‐seq was used to examine transcriptomic changes in tumors after treatment. PCA demonstrated clear separation in sample clustering between the HKL‐nm + DQ‐treated group and the PBS control group, reflecting substantial transcriptomic modifications induced by HKL‐nm + DQ therapy (Figure 6h). We identified 155 genes that were upregulated and 245 genes that were downregulated (Figure 6i,j). To further understand the biological implications of these differentially expressed genes (DEGs), we performed KEGG pathway enrichment analyses. The findings indicated significant enrichment in immune‐related pathways, such as “T cell receptor signaling pathway,” “natural killer cell mediated immunity,” “positive regulation of T cell activation,” “T cell proliferation,” and “immune response‐activating signal transduction” (Figure 6k). These pathways are strongly linked to heightened immune responses, which corroborates the mechanism by which HKL‐nm + DQ enhances tumor suppression through immune modulation.

## 3 DISCUSSION

The development of effective chemotherapeutic approaches for HCC continues to face formidable obstacles, stemming from the tumor's inherent biological aggressiveness, high post‐treatment recurrence rate, and a profoundly immunosuppressive TME. Here, we present a nanotherapeutic platform based on HKL‐nm, which overcomes the pharmacokinetic and bioavailability limitations of free HKL. Our findings demonstrate that HKL‐nm stabilizes mtDNA and inhibits the cGAS‐STING pathway, while combination with the DQ senolytic regimen enhances cytotoxic T cell infiltration and alleviates myeloid‐derived immunosuppression. By integrating nanotechnology, senescence biology, and immuno‐oncology, this study establishes a transformative paradigm for HCC treatment.

By encapsulating HKL within a biodegradable PEG‐PCL‐PEG triblock copolymer nanomicelle, we achieved a spherical architecture with a narrow size distribution (60.93 ± 5.7 nm) and near‐neutral zeta potential, enhancing colloidal stability and minimizing non‐specific interactions. Our prepared HKL‐nm exhibit a nearly neutral surface potential (−0.28 ± 0.1 mV), which is primarily attributed to the dense PEG corona shielding the charge of the inner hydrophobic core. This PEGylation strategy not only ensures the colloidal stability of the micelles in circulation by providing steric hindrance but is also designed to reduce the non‐specific adsorption of opsonins, minimizing non‐specific interactions with plasma proteins and other biological components. This allows the nanoparticles to “disguise” themselves and evade rapid recognition and clearance by the Mononuclear Phagocyte System (MPS).^16^, ^17^ Our pharmacokinetic study results (Figure 2e,f) provide evidence for the success of our design; compared to free HKL, HKL‐nm demonstrated a significantly prolonged plasma half‐life (a 6.26‐fold increase in AUC) and enhanced accumulation at the tumor site by 3.52‐fold (Figure 2g). These findings confirm that our PEGylated nanomicelles successfully achieved the intended goals of extended systemic circulation and improved tumor targeting. These improvements align with prior studies demonstrating that PEGylated nanocarriers prolong systemic circulation and reduce off‐target toxicity by leveraging the enhanced permeability and retention (EPR) effect.^18^ Notably, the negligible systemic toxicity observed in HKL‐nm‐treated mice underscores its clinical translatability.

Cellular senescence plays a dual role in tumor progression and contributes to therapy resistance and immunosuppression via SASP‐mediated TME remodeling.^19^, ^20^ Our work demonstrates that HKL‐nm effectively suppresses senescence in HCC cells, reducing SA‐β‐gal‐positive populations accompanied by downregulation of p53, p16, and p21. A key innovation of this study is the identification of a SIRT3‐dependent mechanism by which HKL‐nm stabilizes mtDNA and inhibits the cGAS‐STING pathway. mtDNA leakage into the cytoplasm activates cGAS‐STING, driving SASP and immune evasion.^21^, ^22^ HKL‐nm treatment reduced cytoplasmic mtDNA fragments (16S rRNA, D‐loop, and Nd4) and prevented phosphorylation of cGAS, STING, and IRF3. Critically, SIRT3 knockdown abrogated these effects, confirming that mitochondrial integrity is maintained via SIRT3‐mediated mechanisms. Furthermore, the knockdown of SIRT3 expression inhibits the effects of HKL‐nm. Our results align with the findings reported in the published literature, specifically that HKL, as an agonist of SIRT3, primarily exerts its effects through the activation of SIRT3.^10^ This represents a novel mode of action for HKL in HCC, distinct from its known regulation of NF‐κB and Ras–RAF pathways,^23^, ^24^ and establishes a link between mitochondrial biology, senescence, and immune modulation. This SIRT3‐dependent mechanism represents a novel therapeutic axis, as prior studies have predominantly focused on direct cGAS‐STING inhibitors rather than mitochondrial stabilization.^25^ HKL‐nm suppressed the SASP, reducing IL‐6 and IL‐8 secretion by ~35% in HepG2 cells. RNA‐seq analysis further identified broad suppression of SASP‐related genes, including pro‐inflammatory cytokines (IL1α and IL1β) and MMP4. The observed suppression of IL‐6, IL‐8, and pro‐inflammatory chemokines further corroborates HKL‐nm's ability to attenuate SASP, thereby disrupting the feedforward loop between senescence and immune evasion.

The combination of HKL‐nm with the senolytic regimen DQ (dasatinib + quercetin) yielded striking synergistic benefits. While HKL‐nm monotherapy reduced tumor volume by 67% in xenograft models, the combination further decreased volume by 81%, accompanied by enhanced apoptosis (cleaved caspase‐3 upregulation) and suppressed proliferation (Ki‐67 reduction). This synergy likely arises from complementary mechanisms: HKL‐nm inhibits senescence induction, while DQ eliminates residual senescent cells, collectively reducing SASP‐mediated immunosuppression.^26^, ^27^ Flow cytometry revealed that the combination increased tumor‐infiltrating CD8^+^ T cells by 4.6‐fold and mature dendritic cells (DCs) by 3.9‐fold, while MDSCs increased by 72%. These changes align with RNA‐seq findings showing enrichment of immune‐related pathways, demonstrating that HKL‐nm + DQ reprograms the TME into an immunogenic state. Our RNA‐seq data also revealed significant enrichment of immune‐related pathways, including cytokine signaling and T cell activation, in HKL‐nm‐treated tumors. This immunogenic reprogramming, coupled with the absence of systemic toxicity, positions HKL‐nm as a promising candidate for clinical translation. These findings align with emerging evidence that senolysis potentiates immune checkpoint blockade,^28^ and our study uniquely integrates natural product‐based nanotherapy with senolytic regimens, offering a safer and more scalable approach.

However, several questions remain. First, a comparative uptake study with free HKL is a subject for future investigation. This will include designing low‐temperature inhibition experiments to explore whether the uptake of HKL‐nm is energy‐dependent and using specific endocytic pathway inhibitors to elucidate the uptake mechanism. The long‐term effects of mitochondrial stabilization on tumor recurrence warrant investigation, as SIRT3 may exhibit context‐dependent roles in cancer metabolism.^29^ Second, while our xenograft model recapitulates key aspects of HCC, future studies should validate these findings in immunocompetent models or patient‐derived organoids to account for human‐specific immune interactions. The use of subcutaneous xenograft models rather than orthotopic HCC models and the need for further investigation into long‐term biodistribution and toxicity. Finally, optimizing the dosing schedule of HKL‐nm and DQ could further enhance therapeutic efficacy while minimizing potential off‐target effects. Future studies could explore tumor‐targeted modifications (e.g., ligand‐conjugated nanomicelles) to enhance specificity and evaluate combinations with immune checkpoint inhibitors.

## 4 MATERIALS AND METHODS

### 4.1 Materials

All chemicals were analytical grade and acquired from commercial sources. Poly(ethylene glycol) methyl ether (PEG, Mn = 5000), ε‐caprolactone (ε‐CL), and stannous octoate (Sn(Oct)_2_) were used as supplied by Sigma‐Aldrich. HKL was purchased from AK Scientific Inc. (Union City, CA, USA). FBS and Dulbecco's Modified Eagle Medium (DMEM) were obtained from Gibco (Thermo Fisher Scientific, Waltham, MA, USA) and used according to manufacturer specifications.

### 4.2 Cell lines and animals

The hepatoma cell lines HepG2 (#HB‐8065, Homo sapiens, Liver, Male) and Hep3B (#HB‐8064, Homo sapiens, Liver, Male), along with the mouse hepatoma cell line Hepa1‐6 (#CRL‐1830, Mus musculus, Liver, Male) were purchased from the American Type Culture Collection (ATCC, Manassas, VA, USA). Additionally, the human hepatoma cell line Huh7 (#CBP60202, Homo sapiens, Liver, Male) was acquired from Nanjing Cobio Biotechnology Co., Ltd. All cell lines were cultured in DMEM medium containing 10% FBS, and maintained under standard incubation conditions: 37°C, 5% CO_2_, and regulated humidity. Authentication of each cell line was completed before acquisition. These four cell lines were selected based on their widespread application as representative models in studies of HCC. Before initiating experiments, routine mycoplasma testing was conducted using the MycoGuard™ Mycoplasma PCR Detection Kit (Genecopoeia #MP004), and all cultures were verified to be free of mycoplasma contamination.

To generate the therapy‐induced senescence (TIS) model, HepG2, Hep3B, and Huh7 cell lines were treated with 250 nM doxorubicin for 24 h, followed by incubation in drug‐free culture medium for 5–7 days. Senescence was assessed through the presence of typical morphological alterations and confirmed by positive staining for SA‐β‐gal. Additionally, Western blot analysis verified the senescent state by demonstrating increased protein levels of p16, p53, and p21.

Healthy C57BL/6J mice were obtained from Hubei BIONT Biological Technology Co., Ltd. (Wuhan, China) and housed under specific pathogen‐free (SPF) conditions in a laminar airflow cabinet, with controlled environmental parameters including a 12‐h light–dark cycle and a temperature maintained at 25 ± 2°C. All animal procedures were approved by the Animal Care and Ethics Committee of Tongji Hospital (No. TJ‐C20200155).

### 4.3 Preparation of honokiol nanomicelles

In this procedure, PEG served as the macroinitiator and Sn(Oct)_2_ as the catalyst. A precisely measured mixture containing ε‐CL monomer (2 g), PEG macromer (2 g), and Sn(Oct)₂ catalyst (0.01 mmol) was polymerized at 120°C for 12 h under an inert atmosphere. After polymerization, the viscous product was cooled to room temperature, purified by dissolution in chloroform (50 mL) followed by precipitation with ice‐cold diethyl ether (−20°C), and subsequently vacuum‐dried at 25°C for 24 h to remove residual solvents. To prepare HKL‐loaded micelles, PEG‐PCL‐PEG copolymer (20 mg) and varying amounts of HKL (0, 1, 2, 5, 10, 15, and 20 mg) were dissolved in 2 mL of anhydrous acetone. This solution was added dropwise to 25 mL of deionized water using a syringe pump (0.5 mL/min) under vortex mixing (800 rpm). The mixture was then stirred magnetically for 24 h at 25°C to allow solvent evaporation and micelle maturation. Unencapsulated HKL was removed by filtration through a 0.45 μm cellulose acetate membrane. The micelles were collected by centrifugation (20,000 × g, 20 min, 4°C) and subsequently lyophilized for 48 h (−80°C, 14 Pa) to obtain a powder.

### 4.4 Physicochemical characterization of HKL‐nm

Colloidal characteristics, including hydrodynamic diameter and zeta potential (surface charge), were determined by DLS using a Zetasizer Nano ZS (Malvern Panalytical, UK). Morphology was examined by high‐resolution transmission electron microscopy (HR‐TEM) using a Hitachi H‐6009IV microscope (Japan) at an acceleration voltage of 120 kV. Samples were prepared by drop‐casting the micellar dispersion onto carbon‐coated copper grids, followed by negative staining with 2% phosphotungstic acid.

### 4.5 Determination of drug‐loading and encapsulation efficiency

Quantitative determination of drug‐loading parameters followed rigorous protocol: Lyophilized nanoparticles (1.0 mg) were accurately weighed, reconstituted in 1 mL of deuterated chloroform, and centrifuged at 15,000 × g for 10 min to remove polymer interference. The supernatant was analyzed by HPLC as described below. Drug loading capacity (DL%) and encapsulation efficiency (EE%) were calculated using the following equations:

$$\mathrm{DL}\%=\frac{\text{Mass of drug in micelles}\;}{\text{Total mass of micelles}}\times 100.$$

$$\mathrm{EE}\%=\frac{\text{Mass of drug in micelles}}{\text{Initial mass of drug added}}\times 100.$$

Here, *M*_drug_ represents the encapsulated drug mass, *M*_polymer_ denotes copolymer mass, and *M*_initial_ corresponds to the initial drug input.

HPLC analysis was conducted using an Alliance 2695 system (Waters Corp., Milford, MA, USA) fitted with an auto‐sampler kept at 10°C and a SunFire™ C18 column (150 mm × 4.6 mm, 5 μm) held at a temperature of 28 ± 0.5°C. The separation was achieved isocratically using a mobile phase composed of acetonitrile and water in a ratio of 60:40 (v/v) at a flow rate of 1.0 mL/min. A calibration curve was generated across a concentration range of 0.1–50 μg/mL, yielding a correlation coefficient (*R* ^2^) greater than 0.999.

### 4.6 In vitro drug release study

HKL‐nm (0.5 mL, 1 mg/mL HKL) and free HKL in DMSO (0.5 mL, 1 mg/mL) were loaded into prehydrated dialysis cassettes (MWCO 3.5 kDa; surface area 1 cm^2^). Cassettes were immersed in 10 mL of release medium (PBS pH 7.4 with 0.5% Tween 80) at 37 ± 0.5°C under agitation (100 rpm) in a USP‐compliant dissolution apparatus. At predetermined intervals (0, 0.5, 1, 2, 5, 10, 20, 50, 70, 80, 90, 120, 150, and 200 h), 0.5 mL aliquots were withdrawn and immediately replenished with equivalent volumes of pre‐equilibrated fresh medium. Quantitative analysis was performed through a validated HPLC method as previously described. Cumulative drug release was calculated.

### 4.7 Cellular uptake

HepG2 cells were plated at 2 × 10^5^ cells/well in μ‐Dish 35 mm glass‐bottom confocal plates (Ibidi, Germany) and maintained under standard culture conditions (5% CO_2_, 37°C) for 24 h to achieve 80% confluency. For time‐dependent cellular internalization studies, cell cultures were exposed to Dil‐fluorescent HKL‐nm (25 μg/mL) for incubation periods of 2, 6, 12, 24, and 48 h. Three experimental groups were incorporated: a PBS control group, a blank nanomicelle (PEG‐PCL‐PEG) group labeled with Dil dye, and a drug‐loaded nanomicelle (HKL‐nm) group also labeled with Dil. Post‐treatment, cells underwent triple‐rinsing with ice‐cold PBS (pH 7.4) followed by nuclear counterstaining with 4′,6‐diamidino‐2‐phenylindole (DAPI, 10 μg/mL in PBS) for 10 min at 25°C protected from light. Spatiotemporal localization of nanocarriers was visualized using a Leica TCS SP8 STED super‐resolution confocal microscope (Leica Microsystems, Germany) equipped with 405 nm (DAPI) and 549 nm (Dil) laser lines. Quantitative internalization profiles were obtained via BD FACSymphony™ A5 flow cytometer (BD Biosciences, USA) using 549 nm excitation. Fluorescence intensity from 10,000 individual cell events was analyzed using FlowJo v10.8 software, with data normalized to untreated controls.

### 4.8 In vitro cytotoxicity assessment

HepG2 cells were treated with various concentrations of HKL‐nm or blank nanomicelles, and cells treated with PBS served as the control. HepG2 cells were plated at a density of 1.0 × 10^4^ cells per well in 96‐well plates (Corning Costar, USA) with 100 μL of complete culture mediu. After a 24‐h incubation under optimal growth conditions to reach 80% confluency, the cells were exposed to varying concentrations (1, 5, 10, 20, 50, and 100 μg/mL) of polymeric micellar formulations for an additional 24 h. Following treatment, cell viability was assessed using the colorimetric Cell Counting Kit‐8 (CCK‐8, Wuhan, Biosharp Life Science) assay following the manufacturer's instructions.

### 4.9 In vivo safety evaluation

The toxicological profile of HKL‐nm was comprehensively evaluated in male C57BL/6J tumor‐bearing mice (8 weeks old, weighing 22 ± 2 g) through multimodal safety monitoring. The in vivo safety of HKL‐nm was assessed after both short‐term (24 h) and long‐term (28 days) administration. Mice treated with saline were used as the control group. For the short‐term evaluation: Healthy C57BL/6J mice were randomly allocated into two groups, receiving a single dose of either saline or a relatively high dose of HKL‐nm (100 mg/kg). One day after the treatment, the mice were humanely euthanized, and organ and blood samples were collected and analyzed. The main organs, namely the heart, liver, spleen, lungs, and kidneys, were harvested and processed for H&E staining to detect possible structural alterations. The blood parameters analyzed included white blood cell count (WBC), lymphocyte count (LYM), monocyte count (MON), and neutrophil count (GRAN). Aspartate aminotransferase (AST), alanine aminotransferase (ALT), creatinine (CRE), and blood urea nitrogen (BUN) were measured using commercially available liver and kidney function test kits. Regarding the measurement of inflammatory factor levels, inflammatory cytokines, such as TNF‐α, IL‐1β, and IL‐6, were quantified using ELISA assays according to the manufacturer's protocols. Long‐term: Healthy C57BL/6J were randomly assigned into two groups, receiving either saline or HKL‐nm (40 mg/kg) once every 7 days for a total of four treatments. Mice were euthanized 1 day after 28 days, and major organs (heart, liver, spleen, lungs, and kidneys) as well as blood samples were collected and analyzed as described above.

### 4.10 Oral pharmacokinetic profiling

Comparative bioavailability studies were conducted using male C57BL/6 mice (8 w, 25 ± 2 g). The experimental design comprised two arms: free HKL suspension (100 mg/kg) and HKL‐nm (equivalent dose) administered via oral gavage using 20G stainless steel feeding needles. The animals treated with free HKL served as the control. Animals were randomized (*n* = 5–8 per group) with a staggered euthanasia protocol. Serial blood specimens (300 μL) were collected into K2EDTA‐coated microtainers (BD Biosciences) at 0.25, 0.5, 1, 2, 4, 6, 8, 12, 18, 24 h post‐administration. Plasma separation was achieved through dual centrifugation (3000 × g, 10 min at 4°C followed by 10,000 g, 5 min) using a refrigerated centrifuge (Eppendorf 5430R). Plasma samples underwent extraction using the protein precipitation method, and the concentration of HKL was determined through HPLC. The analysis of pharmacokinetic data was conducted according to established procedures, assessing parameters including the AUC, the peak plasma concentration (*C*_max_), and additional relevant metrics.

### 4.11 Senescence‐associated β‐galactosidase assay

HepG2 cells were subjected to SA‐β‐gal staining using an SA‐β‐gal staining kit (Beyotime Biotechnology, China; #C0602) according to the manufacturer's protocol. Specifically, the cells were fixed with 4% paraformaldehyde for 5 min. After being washed three times with PBS, the samples were incubated in the SA‐β‐gal staining solution at 37°C for 16–18 h. The reaction was halted with ice‐cold PBS. In blinded evaluations, 10 random microscopic fields were imaged for each sample. The proportion of positive cells was determined by counting the blue‐stained cells and dividing this number by the total number of cells present.

### 4.12 Measurement of IL‐6 and IL‐8 concentrations by ELISA

The levels of IL‐6 and IL‐8 were measured using ELISA kits (R&D Systems) with slight modifications. Cell culture supernatants were harvested after centrifugation at 1000 g for 10 min at 4°C. Precoated 96‐well plates were equilibrated to room temperature. Standards (0–500 pg/mL for IL‐6, 0–1000 pg/mL for IL‐8) and samples (50 μL per well) were added in duplicate and incubated for 2 h at 25°C. Plates were washed five times with PBST, followed by incubation with biotinylated antibodies (50 μL per well) for 1 h. After washing, streptavidin‐HRP conjugate (50 μL per well) was added and incubated for 20 min. Color development was achieved with TMB substrate (100 μL per well), stopped with 2 N H_2_SO_4_, and absorbance was measured at 450 nm (reference 570 nm) using a BioTek Synergy H1 reader.

### 4.13 Anticancer effects of HKL‐nm in cancer xenograft models

For in vivo anticancer studies, 1 × 10^6^ Hepa1‐6 cells (100 μL) were subcutaneously injected into the right flank of 6‐ to 8‐week‐old C57BL/6J mice. One week following the injection of tumor cells, palpable tumors became evident, confirming successful tumor formation. Tumor volumes were estimated using the formula: tumor volume (mm^3^) = (width (mm)^2^ × length (mm))/2. Upon reaching a volume of approximately 100 mm^3^, the mice were allocated into four experimental groups. The groups were treated as follows: PBS, free HKL (40 mg/kg, administered via tail vein injection on a weekly basis), HKL‐nm (nanomicellar formulation, 40 mg/kg, administered via tail vein injection on a weekly basis), and HKL‐nm + DQ (Dasatinib + Quercetin; Dasatinib at 5 mg/kg and Quercetin at 100 mg/kg, administered intraperitoneally three times per week). Treatment lasted 4 weeks, with weekly monitoring of tumor volumes and body weights. At the end of treatment, animals were euthanized, and final tumor volumes and weights were measured. Tumors were analyzed by western blot and immunohistochemistry, and samples were prepared for TUNEL assay and Ki‐67 staining.

### 4.14 Western blot analysis

Tumor tissues were homogenized in ice‐cold RIPA buffer containing 1% protease inhibitor and 1 mM PMSF. For cell samples, whole‐cell proteins were extracted by lysing cells on ice for 30 min in a buffer with 10 mM Tris–HCl (pH 7.5), 150 mM NaCl, 1% NP‐40, 1 mM EDTA, and 1 mM PMSF. Lysates were centrifuged at 12,000 × g for 15 min at 4°C, and supernatants were collected. Protein concentrations were measured using the BCA assay. Equal protein amounts (20 μg) were separated by SDS‐PAGE and transferred onto nitrocellulose membranes (0.45 μm pore size) at 300 mA for 90 min. Membranes were blocked with 5% non‐fat milk in TBST for 1 h at room temperature, incubated with primary antibodies overnight at 4°C, and then washed three times with TBST. HRP‐conjugated secondary antibodies (1:5000 dilution) were applied for 1 h at room temperature. Protein bands were detected using ECL Plus and documented on autoradiography films. Band intensities were quantified using densitometry with the Bio‐Rad Chemi‐Doc system and further analyzed semi‐quantitatively with ImageJ. Primary antibodies are listed in the figure legends.

### 4.15 Real‐time quantitative polymerase chain reaction analysis

Gene expression analysis specific to the target genes was performed using real‐time quantitative polymerase chain reaction (RT‐qPCR). In summary, total RNA was isolated employing QIAzol Lysis Reagent and RNeasy Mini Columns (Qiagen, Valencia, CA, USA). The quality and purity of the RNA samples were assessed with a NanoDrop spectrophotometer (Thermo Scientific, Wilmington, DE, USA). Synthesis of the first‐strand cDNA was conducted using the RevertAid First Strand cDNA Synthesis Kit (Thermo Scientific, Waltham, MA, USA; Catalog #K1622). Real‐time PCR amplifications were carried out on the ABI Prism 7900HT System (Applied Biosystems, Carlsbad, CA, USA) with SYBR Green Master Mix (Roche Applied Science, Basel, Switzerland; Catalog #04913850001). The primer sequences utilized in this study are provided in the Table 1.

**TABLE 1:** Primers for real‐time quantitative polymerase chain reaction.

	Forward	Reverse
16S rRNA	CCGCAGGTTCACCTACGGGAG	CGCCCGTCAGTTCCGATTACT
TFAM	GCTGCTGAAGATGGTGAAGGTC	AGGCTTGCTGTTCTTCTCGTCT
Nd4	TGGCCTATGGTGTTTGTGGTG	CAGGCTGTTGGTTGTAGGTGA
D‐loop	CCTCCGATCTAGTATCACCACCATC	GAGGGTGAGGGTGTTGTAGTAG
18S rRNA	CGGAATTGGACAGATGATTGAC	GCTGGAATTACCGCGGCT

### 4.16 In vivo immune response analysis

To study the immune response to the treatments, tumor tissues and lymph nodes were harvested, processed into single‐cell suspensions, and prepared as follows: Samples were cut into small pieces, digested with collagenase IV (1.0 mg/mL), hyaluronidase (0.2 mg/mL), and deoxyribonuclease (0.1 mg/mL) at 37°C, lysed of red blood cells, and filtered through a 200‐mesh nylon mesh. Cells were pelleted by centrifugation at 1000 rpm for 3 min, washed with PBS containing 2% FBS, and resuspended for analysis. Single‐cell suspensions were stained with fluorescently conjugated flow cytometry antibodies targeting membrane surface markers following standard protocols. Mature DCs were identified using the Zombie NIR Fixable Viability Kit, PerCP‐CD45, APC‐CD11c, FITC‐CD80, and PE‐CD86. Additionally, T cells were labeled with the Zombie NIR Fixable Viability Kit, PerCP‐CD45, BV421‐CD3, PE‐CD8, FITC‐TNF‐α, and APC‐IFN‐γ antibodies. MDSCs were stained with the Zombie NIR Fixable Viability Kit, PerCP‐CD45, FITC‐CD11b, and BV605‐GR‐1 antibodies. All stained single‐cell suspensions were analyzed using a Beckman flow cytometer (USA).

### 4.17 RNA‐sequencing analysis

Total RNA was extracted from the samples using the RNeasy Mini Kit (Qiagen, Germany). Libraries were constructed with the TruSeq™ RNA Sample Preparation Kit (Illumina, USA). Polyadenylated mRNA was isolated using poly‐T oligo‐attached magnetic beads. The mRNA was fragmented at 94°C for 8 min in a divalent cation buffer. First‐strand cDNA synthesis used random hexamers and reverse transcriptase, followed by second‐strand synthesis with DNA polymerase I and RNase H. Double‐stranded cDNA underwent end repair, adenylation, adapter ligation, and PCR amplification. Library quality was assessed using the Qubit® 2.0 Fluorometer (Thermo Scientific, USA) and Agilent 2100 Bioanalyzer (Agilent Technologies, USA). Normalized libraries (10 pM) were sequenced on the Illumina HiSeq 2500 system (Illumina, USA), generating paired‐end reads (2 × 150 bp) with an average depth of 40 million reads per sample. Raw sequencing data were evaluated using FastQC (v0.11.9). Adapters were removed and quality filtering was performed with Trimmomatic (v0.39) using parameters: SLIDINGWINDOW:4:20, MINLEN:36. Reads were mapped to GRCh38.p13 with STAR (v2.7.9a), achieving >85% mapping efficiency. Transcript quantification used featureCounts (v2.0.3) with GENCODE v35 annotations. Differential expression analysis was conducted in R (v4.2.1) using DESeq2 (v1.38.3), applying Benjamini–Hochberg correction (FDR <0.05). Genes with |log2(fold change)| >1 and adjusted *p*‐value <0.05 were considered significantly differentially expressed. Functional enrichment analysis was performed with clusterProfiler (v4.6.2) using GO biological processes and KEGG pathways. Protein–protein interaction networks were reconstructed with STRING (v11.5) and visualized with Cytoscape (v3.9.1). Batch effects were corrected using Combat‐seq (sva v3.46.0) when necessary. Quality assessments included PCA (factoextra v1.0.7) and sample correlation heatmaps (pheatmap v1.0.12). All analyses included three biological replicates per condition.

### 4.18 Statistical analysis

All experimental outcomes were presented as the mean ± standard deviation (SD). Statistical evaluations were carried out using GraphPad Prism 10 software. In cases of comparing two groups, Student's *t*‐tests were utilized, whereas one‐way analysis of variance (ANOVA) was applied for comparisons involving multiple groups. The criteria for statistical significance were set as follows: *p < 0.05, **p < 0.01, ***p < 0.001, ****p < 0.0001.

## 5 CONCLUSION

In summary, this study establishes HKL‐nm as a nanotherapeutic that addresses the pharmacokinetic challenges of HKL while targeting senescence and immune evasion in HCC. By elucidating the SIRT3‐mtDNA‐cGAS‐STING axis as a central mechanism, we provide a strategy to combine natural products, nanotechnology, and senolysis for combating cancers. These findings not only advance our understanding of senescence‐immune crosstalk but also pave the way for clinical trials evaluating HKL‐nm‐based regimens in HCC.

## AUTHOR CONTRIBUTIONS

All authors played a significant role in conceptualizing and designing the study. All authors thoroughly examined the manuscript and actively participated in its extensive revision before submission. All authors have carefully perused and approved the final version of the manuscript. Methodology, investigation, formal analysis, writing—original draft, and writing—review and editing by **WD**. Conceptualization, investigation, formal analysis, and writing—original draft by **YW**. Methodology, investigation, and writing—original draft by **YY**. Supervision, project administration, and writing—review and editing by **JW**.

## CONFLICT OF INTEREST STATEMENT

The authors declare no conflict of interest.

## Supporting information


**Data S1.** Supporting Information.

## Data Availability

All data generated or analyzed during this study are included in this article and its Supporting Information S1. Further inquiries can be directed to the corresponding author (Jing Wang, wangjing3962@tjh.tjmu.edu.cn).
