# Supplementary material for: Honokiol‐loaded nanomicelles reprogram senescence and immune evasion in hepatocellular carcinoma via SIRT3‐mediated mitochondrial stabilization
Source: Bioeng Transl Med. 2026 Jan 18;11(3):e70111. doi: 10.1002/btm2.70111 (PMC13247405; doi:10.1002/btm2.70111)
Supplement: Supplementary file 1 — Data S1. Supporting Information. [file BTM2-11-e70111-s001.docx]

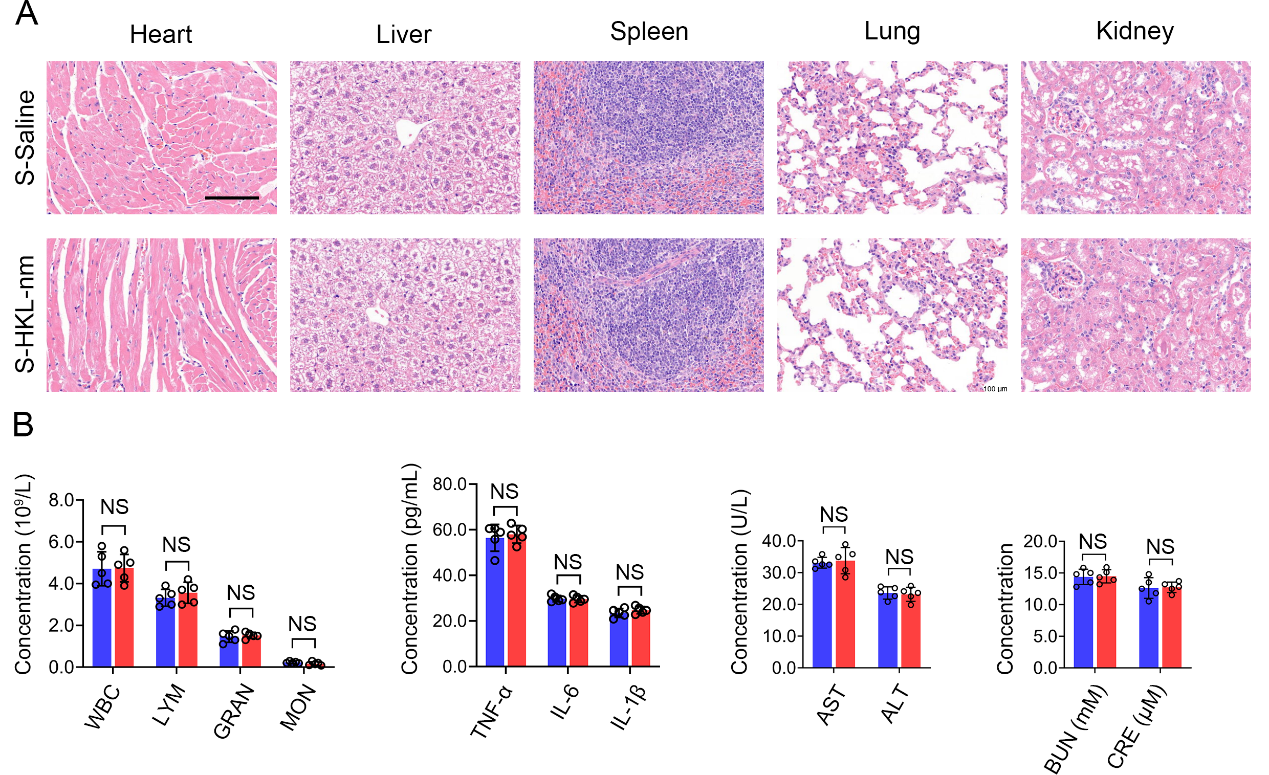


Supplementary Figure S1. HKL-nm exhibited excellent biocompatibility. (A) Representative H&E stained images of the major organs from mice following short - term administration of HKL-nm or saline. (B) Hematological parameters, inflammatory cytokine levels liver function indicators, and kidney function indicator of the short - term treated mice in different treatment groups. Data are presented as mean ± SD. n = 5. White Blood Cell Count (WBC), Lymphocyte Count (LYM), Neutrophil Count (GRAN), Monocyte Count (MON), Aspartate Aminotransferase (AST), Alanine Aminotransferase (ALT), serum creatinine (CRE), blood urea nitrogen (BUN).

Supplementary Figure S
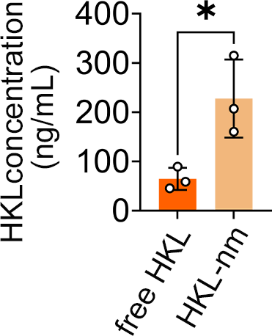
2. HKL concentration in tumor tissue.*p < 0.05.
